# Quantitative evaluation of essential medicines lists: the South African case study

**DOI:** 10.1186/s12913-016-1937-x

**Published:** 2016-12-12

**Authors:** Velisha Ann Perumal-Pillay, Fatima Suleman

**Affiliations:** Discipline of Pharmaceutical Sciences, School of Health Sciences, Westville Campus, University of KwaZulu-Natal, Private Bag X54001, Durban, 4000 South Africa

**Keywords:** Essential medicines, Essential medicines lists, Priority medicines, Standard treatment guidelines, Health systems research

## Abstract

**Background:**

The South African (SA) health system has employed an Essential Medicines List (EML) with Standard Treatment Guidelines (STGs) since 1996. To date no studies have reported the changes in SA STG/EMLs. This study describes these changes over time (1996–2013) and compares latest SA STG/EMLs with the latest World Health Organization (WHO) Model EMLs to assess alignment of these lists.

**Methods:**

A quantitative evaluation of SA STGs/EMLs at 2 levels of healthcare was performed to assess changes in the number and ratio of molecules, dosage forms, and additions and deletions of medicines. The most recent WHO EMLs (18th list, 4th list for children) and 2012 priority life-saving medicines for women and children (PMWC) list were compared to the most recent available SA STG/EMLs (Primary Health Care (PHC 2008), Adult Hospital 2012, and Paediatric Hospital 2013) at the time of the research.

**Results:**

The number of molecules over the years increased for PHC STG/EMLs but decreased slightly for Adult and Paediatric hospital STG/EMLs. The most additions and deletions over time occurred in the Adult hospital level STG/EML (27 in 2006 and 44 in 2012). A comparison between the most recent SA STG/EMLs and WHO Model EML (18th list) showed that a total of 112 medicines were absent on all SA STG/EMLs. A comparison of medicines for children between the 2013 SA Paediatric Hospital level STG/EML and PMWC indicated that these lists were somewhat aligned for most conditions as only 3 of 14 medicines and 11 of 20 vaccines were absent from SA STG/EMLs.

**Conclusion:**

This is the first study in SA to investigate changes in National EMLs over time in relation to molecules, dosage forms and therapeutic classes. It is also the first to compare the latest SA STG/EMLs to the WHO Model lists. The results therefore provide insight into the trends and SA STG/EML processes over time.

## Background

The essential medicines concept is recognized worldwide as a tool to improve health equity and to promote the cost-effective use of health resources. It is vital in countries with limited resources to not only manage health services but also to provide guidance on the best way to maximize available resources. It has been established that the judicious selection of a limited number of essential medicines results in improved medicine management and enhanced quality of care. The Essential Medicines List (EML) is a fundamental tool which guides countries in the procurement and distribution processes, and which ultimately reduces costs to both the health care system and the patient [1, 2].

In 1977, the World Health Organization (WHO) developed a Model EML which is updated every 2 years. It is a tool to guide countries to develop their own National EML (NEML) [3].

The WHO defines essential medicines as

“those that satisfy the priority health care needs of the population. These are selected with regard to public health relevance, evidence on efficacy and safety, and comparative cost-effectiveness; and should be available and affordable at all times within functioning health systems. Countries then determine which medicines are considered essential” [4].

In April 2011, WHO published a list of Priority Medicines for Mothers and Children, which was revised in 2012 to Priority life-saving medicines for women and children (PMWC). It is a subset of medicines from the WHO EML developed by experts in the field of maternal and child health. These medicines were selected taking into account the worldwide disease burden and evidence of safety and efficacy. Priority conditions identified by WHO include: HIV/AIDS, malaria, pneumonia, tuberculosis and diarrhoea for children, and for mothers: post-partum haemorrhage; pre-eclampsia, sepsis, sexually transmitted infections and preterm birth [5].

South Africa (SA) has implemented an EML with Standard Treatment Guidelines (STG) since 1996. This was a key objective of the 1996 National Drug Policy for South Africa (NDP) to ensure equity in health care for all citizens after the fragmentation that was created under apartheid [6].

Although the under-5 years mortality rate in South Africa has decreased in recent years, from 60 deaths per every 1 000 live births in 1990 to 41 in 2015, it still failed to meet the target (20) set out in the Millennium Development Goals (MDG) [7]. Ensuring access to simple and affordable medicines, such as oral rehydration for diarrhoea could prevent death in children [8]. These medicines including PMWC could be included in the SA STG/EMLs and will contribute to achieving the new proposed Sustainable Development Goals (SDGs) target for child mortality by 2030. The target is to end preventable deaths of new-borns and children under five years and for countries to decrease neonatal mortality to 12 deaths per 1000 live births and children under-5 years mortality to 25 deaths per 1000 live births [7].

This PMWC list was selected as it reflects WHO’s recommendation for the most important medicines for mothers and children and according to Hill et al., (2012) should be part of NEMLs. NEMLs are key policy tools for promoting the supply of priority medicines, especially in the public sector [9]. The regular production, updating and maintenance of NEMLs, that is evidence based and transparent, is vital for the successful implementation of a national Essential Medicines Programme (EMP) [10, 11]. SA still has a two-tiered healthcare system encompassing both public and private sectors. The public sector has three levels of care (primary health care (PHC), Hospital level secondary and tertiary/quaternary level. There are 3 STG/EML publications, for PHC, adult hospital and paediatric hospital care. The listing of medicines for tertiary/quaternary care is quite different; less detailed, and does not include STGs. The Tertiary and Quaternary Level EMLs were published in November 2012 [12] but are not included in this study as it is a list of recommendations for use of medicines for certain indications.

The PHC EML has been revised four times (editions in 1996; 1998; 2003; 2008; 2014); the Adult Hospital level revised three times (1998; 2006; 2012; 2015) and Paediatric hospital level revised twice (1998; 2006; 2013). WHO recommends regular revision of lists to ensure the selection of medicines remains credible, current, and relevant to the healthcare needs of the population and the country’s budget for health care [11]. However, these lists should not be reviewed too quickly as implementation and acceptance is slow within health systems due to challenges of alignment of procurement processes with the list [13]. The National Essential Medicines List Committee (NEMLC) is responsible for compiling and revising STGs and/or EMLs for each level.

The SA EMLs are derived from the national STGs. The STGs are presented in chapters which are organized according to the organ systems of the body with component topics in alphabetical order. Each guideline outlines the non-pharmacological management followed by the recommended medicines and dosages per disease condition. Medicines are listed as their generic name or International Non-proprietary Name (INN). As the process of medicine selection has evolved, more recent editions of the STGs include the designation of therapeutic classes under the “Medicine treatment” section which lists an example of a medicine within that therapeutic class. Therapeutic classes were designated in cases where none of the members within that class exhibits significant benefit over another. The intention of limiting the listing to therapeutic classes was to promote competition for procurement of medicines to obtain the best possible price during the tender process. The provincial Pharmacy and Therapeutics Committees (PTC) then decides on their medicine of choice within a therapeutic class, based on this tender process, and lists this on their local procurement catalogue [14, 15].

This is similar to the square box symbol used in the WHO Model list of Essential medicines. The square box symbol indicates that the medicine listed is an example of a clinically equivalent medicine from the same pharmacological class. This allows flexibility during the medicine selection process as countries can then select medicines from a class dependent upon current cost and availability [11]. Currently, there is no published information on the changes, process and evaluation of the SA STG/EMLs. This study therefore aims to provide insight into the development of the SA STG/EMLs over time (1996–2013) and to provide a comparative analysis with the latest WHO Model EMLs and PMWC list to assess alignment of these lists.

## Methods

An observational, descriptive study design was employed to quantitatively analyse SA STG/EMLs over time. Hard copies of SA STG/EMLs (1996–2013) were converted into Microsoft EXCEL workbooks for ease of comparisons across years and editions.

SA STG/EMLs were assessed in terms of quantitative changes. The number and ratio of molecules, dosage forms, additions and deletions of medicines per revision were calculated.

The STG/EMLs used in this study were: PHC (1996, 1998, 2003, 2008); Adult hospital level (1998, 2006, 2012) and Paediatric hospital level (1998, 2006, 2013). The SA STG/EML procurement catalogue,^1^ which is a list of tendered medicines as per EML recommendation at that time, was used to complete missing information on dosage forms of a molecule in the NEML [16].

The most recent WHO EMLs (18th list [17]; 4th list for children [18]) and 2012 WHO PMWC list were used to compare the most recent SA STG/EMLs for each level (PHC 2008; Adult Hospital 2012; Paediatric Hospital 2013) for alignment of lists. At the time of this analysis, these were the latest versions of SA STG/EMLs. Both the core [17] and complimentary [17] lists of the WHO Model lists were included in the analysis. Medicines (single molecules or combinations) that did not appear on any of the SA STG/EMLs but were present on the latest WHO Model EMLs were considered absent and recorded in a table. These results reflect the lists for PHC and Hospital levels (Adult and Paediatric).

The term “molecule” refers to the generic name or INN of a medicine and not the different dosage forms listed.

Duplicates are considered as molecules that are listed more than once in the same STG/EML or in different sections of the list. The list of molecules for each STG/EML was analysed in EXCEL using a what-if analysis to flag and identify a molecule that appeared more than once in the same list. Such molecules were assigned a value of 1 which was then totalled to provide the total number of duplicate molecules for that list. This number of duplicates was then subtracted from the total number of molecules for the list to provide the number of unique molecules i.e. the number of molecules per list excluding duplicates. Whilst calculating the total number of duplicates allows for comparison with other reports it is also a reflection of the different ways the lists are arranged. The WHO Model EMLs are arranged according to pharmacological class whilst the SA STG/EMLs are arranged according to organ systems.

Over the years the SA STG/EMLs have been developed to include the listing of therapeutic classes with an example of a medicine within this class as opposed to listing a single preferred molecule as the medicine of choice for a specific indication. Therefore, for this study the number of therapeutic classes listed in each STG/EML was counted and compared across STG/EMLs and across years.

The WHO square box notation to illustrate the listed medicines with therapeutic class alternatives was accommodated for in the comparative analysis of STG/EMLs. When such a box appeared on the WHO EML, the SA STG/EML listing of a molecule in the same class was considered and if present was considered as concordance between the lists. If no therapeutic alternative was listed, the molecule was considered to be absent on the SA STG/EML and subsequently recorded in the table. The comparison was performed for both core and complimentary lists of medicines as listed by WHO.

A year-on-year comparison of molecules was made by comparing the older edition STG/EML with the subsequent issue. This was done for both PHC and Hospital levels. A new appearance of a molecule in the later version STG/EML was considered an addition to the list whilst the omission of a molecule from a later version was considered a deletion.

Ratios of dosage forms per molecule per STG/EML are calculated as: The total number of dosage forms divided by the total number of molecules. The ratios of molecules per organ system classification between the first and latest STG/EMLs are calculated as: The total number of molecules (latest STG/EML) divided by the total number of molecules (First STG/EML). For the ratio calculations a value <1 indicates a decrease and a value >1 indicates an increase.

## Results

### Quantitative changes in the SA STG/EMLs over time

The first PHC STG/EML (1996) contained 180 molecules including duplicates (134 molecules excluding duplicates). The most recent PHC STG/EML (2008) contained 231 molecules including duplicates (161 molecules excluding duplicates), showing an increase in the total number of molecules over the years. Table 1 summarizes these quantitative changes over time for all STG/EMLs.

**Table 1:** Quantitative changes of SA STG/EMLs over time (1996–2013)

Level of SA STG/EML	Year of SA STG/EML	Total molecules (excluding duplicates)	Total Molecules (including duplicates)	Total dosage forms (including duplicates)	Ratio dosage forms per molecule (including duplicates)	Total number of therapeutic classes present
Primary Health Care	1996	134	180	201	1.12	0
	1998	125	177	198	1.12	0
	2003	140	202	219	1.08	6
	2008	161	231	243	1.05	12
Adult Hospital	1998	364	397	447	1.13	0
	2006	390	428	432	1.01	32
	2012	357	395	407	1.03	31
Paediatric Hospital	1998	231	316	368	1.16	1
	2006	240	348	384	1.10	12
	2013	228	342	372	1.09	22

Both Adult and Paediatric hospital STG/EMLs had a slight decrease in number of molecules excluding duplicates from the first lists (1998) to the most recent lists (2012 and 2013 respectively) as a result of using therapeutic classes as an option rather than individual therapeutic molecules for indications. For example, the 1998 Adult list did not contain any therapeutic classes whilst the 2006 and 2012 editions contained 32 and 31 therapeutic classes respectively. The ratio of dosage forms per molecule including duplicates per STG/EML edition decreased over time indicating fewer dosage forms per molecule were listed due to therapeutic classes being nominated.

SA STG/EMLs are arranged in 14 sections classified according to organ systems of the body. Table 2 shows that some sections expanded considerably over time when compared to the others. At PHC level such sections include Blood and Blood-forming products (ratio 2.8); Cardiovascular system (ratio 1.8); Alimentary tract and metabolism (ratio 1.5); Respiratory system (ratio 1.5); general anti-infectives for systemic use (ratio 1.4) and central nervous system (ratio 1.4). At Adult hospital level the ratio increased for the following sections: Sensory organs (ratio 2.0); Blood and blood-forming products (ratio 1.3); respiratory system (ratio 1.3) and cardiovascular system (ratio 1.2). At Paediatric hospital level the Dermatologicals section tripled over time (ratio 3.3) whilst the following sections had a ratio of 1.6: Genitourinary system and sex hormones; General anti-infectives for systemic use; and Respiratory system. A substantial decrease in ratios for antineoplastic and immunomodulating agents at hospital level for both the adult (ratio 0.3) and paediatric (ratio 0.2) was noted.

**Table 2:** Comparison of number of molecules per organ system classification between the first and latest SA STG/EMLs

Section of SA STG/EML	Primary Health Care			Adult Hospital			Paediatric Hospital		
	1996	2008	Ratio	1998	2012	Ratio	1998	2013	Ratio
Alimentary Tract and Metabolism	25	37	1.5	52	49	0.9	50	44	0.9
Blood and Blood-forming Products	4	11	2.8	18	23	1.3	26	33	1.3
Cardiovascular System	12	22	1.8	34	41	1.2	33	26	0.8
Dermatologicals	21	17	0.8	21	17	0.8	7	23	3.3
Genitourinary System and Sex Hormones	18	17	0.9	30	28	0.9	5	8	1.6
Systemic Hormonal Preparations, excluding Sex Hormones	2	2	1.0	15	17	1.1	9	12	1.3
General Anti-infectives for Systemic Use	39	55	1.4	73	83	1.1	62	98	1.6
Antineoplastic and Immunomodulating Agents	0	0	0.0	29	8	0.3	30	5	0.2
Musculoskeletal System	4	4	1.0	11	12	1.1	8	3	0.4
Central Nervous System	21	30	1.4	58	53	0.9	41	41	1.0
Antiparasitic Products	10	10	1.0	9	7	0.8	10	9	0.9
Respiratory System	10	15	1.5	11	14	1.3	11	18	1.6
Sensory Organs	8	9	1.1	14	28	2.0	10	12	1.2
Various	6	2	0.3	22	15	0.7	14	10	0.7

Table 3 summarizes the additions and deletions of medicines per year of revision. The greatest changes over time occurred in the Adult hospital level STG/EML with net changes of 27 and 44 in 2006 and 2012 respectively. Changes in 2006 were due to (i) additions of medicines in the following sections: General anti-infectives for systemic use (28 of which 10 were antiretroviral medicines); central nervous system (17); Dermatologicals (16); Blood and Blood-forming products (14) and the cardiovascular system (14) and (ii) deletion of medicines from the following sections: General anti-infectives for systemic use (19 of which 12 were antibacterials); Antineoplastic and immunomodulating agents (14); Genitourinary System and Sex Hormones (12); Alimentary Tract and Metabolism (13). Additions in the 2012 Adult STG/EML occurred in the following sections: Sensory Organs (12) and General anti-infectives for systemic use (7) and for deletions: Alimentary tract and metabolism (21); Dermatologicals (13); General anti-infectives for systemic use (11); and Antineoplastic and immunomodulating agents (11).

**Table 3:** Summary table of additions and deletions for each revised edition, including duplicates

Level of SA STG/EML	Year of SA STG/EML	Number of additions*	Number of deletions*	Net change
Primary Health Care	1998	46	47	−1
	2003	37	26	11
	2008	60	34	26
Adult Hospital	2006	150	123	27
	2012	47	91	44
Paediatric Hospital	2006	127	104	23
	2013	93	96	−3

*Comparisons are year-on-year made with the previous edition of the STG/EML

For the 2008 PHC STG/EML, the net change of 26 was attributed to additions in the General anti-infectives for systemic use section (21 of which 15 were antiretrovirals) and deletions in the following sections: Alimentary tract and metabolism (8); Antiparasitic Products (7); and Dermatologicals (5). The decentralization of services to PHC, including the provision of antiretroviral therapy [19–21], could be a reason for the addition of 15 antiretrovirals in 2008, which extended anti-retroviral (ARV) management to PHC facilities.

The largest change at the Paediatric hospital level (23) was noted in 2006. Most additions (26) were noted in the General anti-infectives for systemic use section (antibacterials (8); antiretrovirals (6); antimycobacterials (6); vaccines and immunoglobulins (5)); and Alimentary tract and metabolism (21) and most deletions were noted in the following sections: Alimentary tract and metabolism (21); Dermatologicals (13); General anti-infectives for systemic use section (11); central nervous system (11); and Antineoplastic and Immunomodulating agents (10).

### Comparative analysis of SA STG/EMLs with WHO model lists

A direct comparison of EMLs for WHO and SA posed a challenge as the lists are arranged differently. The WHO list reflects all levels of care and is not separated according to levels as with SA STG/EMLs and on the basis of services offered and the competency of the staff at each facility [15].

The comparative analysis of the lists (Table 4: WHO Model EML (18th list) compared with the latest SA STG/EMLs (PHC 2008, Adult hospital 2006, Paediatric hospital 2012) shows the medicines (individual molecules and combinations) present on the WHO Model EML (core and complimentary lists) but absent on all latest SA STG/EMLs. The following sections showed the most number of absent medicines: anti-infective medicines (50); Antineoplastics and Immunosuppressives (20); Immunologicals (11). A total of 112 medicines (79 core and 33 complimentary) were totally omitted from all SA STG/EMLs combined. These differences may be attributed to listing of medicines recommended globally for conditions that may not be applicable in the SA setting (such as the tropical diseases which are not addressed in the SA STG/EMLs for which examples include leishmaniasis and trypanosomiasis) or merely by virtue of medicines not being registered or readily available in SA either as individual molecules or as the listed combinations as is the case with many of the antimalarials (examples include: Amodiaquine; Proguanil; Artesunate; Artesunate + Amodiaquine; Artesunate + Mefloquine).

**Table 4:** Medicines appearing on the WHO Model EMLs but not on the SA STG/EMLs

Section	Name of medicine (core list)	Name of medicine (complementary list)
2. Medicine for pain and palliative care	Docusate sodium	
4. Antidotes and other substances used in poisoning	Potassium ferric hexacyano-ferrate(II)-2H2O Sodium nitrite Sodium thiosulfate	Fomepizole Sodium calcium edetate Succimer,
5. Anticonvulsants/antiepileptics		Ethosuximide
6. Anti-infective medicines	Levamisole (not registered in SA), Niclosamide, Pyrantel, Diethylcarbamazine (not readily available in SA, can be obtained from pharma company), Ivermectin (not registered in SA, can be obtained after MCC approval) Triclabendazole (not available for human use in SA) Azithromycin, Trimethoprim (available in combination with Sulfamethoxazole), Spectinomycin (not available in SA), Clofazimine (available on named patient basis) Ethambutol + Isoniazid Ethambutol + Isoniazid + Rifampicin Rifabutin Flucytosine (available on named patient basis after MCC approval) Griseofulvin Potassium iodide Atazanavir Saquinavir Indinavir Lamivudine + nevirapine + stavudine (FDC) Lamivudine + nevirapine + zidovudine (FDC) Lamivudine + zidovudine (FDC) Emtricitabine + Tenofovir (FDC) Efavirenz + Emtricitabine + Tenofovir (FDC) Oseltamivir Ribavirin, Diloxinide (not available in SA) Miltefosine, Paromomycin (not available in SA) Sodium stibogluconate, Amodiaquine (not available in SA) Artemether (available in combination with lumefantrine) Artesunate (not registered in SA, can be obtained through the IV artesunate access programme on a named patient basis after MCC approval) Artesunate + amodiaquine (not available in SA) Artesunate + mefloquine (not available in SA), Mefloquine (not registered for treatment in SA) Proguanil (not registered for treatment only prophylaxis and in combination with Atovaquone) Sulfadoxine + Pyrimethamine (no longer recommended due to increased resistance) Pyrimethamine Sulfadiazine (available as silver sulfadiazine) Pentamidine (not available in SA, available on named patient basis) Suramin sodium (not readily available in SA, can be obtained on named patient basis) Eflornithine (not readily available in SA, can be obtained on named patient basis) Melarsoprol (not readily available in SA, can be obtained on named patient basis) Nifurtimox (not available in SA)	Capreomycin, Oxaminiquine (not available in SA) Cycloserine (alternative terizidone is listed) p-aminosalicylic acid (PAS), potassium iodide Pegylated interferon alpha (2a or 2b)
8. Antineoplastic and immunosuppresives		*Cytotoxics*: Ciclosporin, asparaginase, calcium folinate, carboplatin, chlorambucil, mercaptopurine, cytarabine, dactinomycin, daunorubicin, dacarbazine, doxorubicin, docetaxel, mercaptopurine, mesna, thioguanine, etoposide, fluorouracil, ifosfamide, paclitaxel, procarbazine, vinblastine,
10. Medicines affecting the blood	protamine sulfate	
12. Cardiovascular medicines		Sodium nitroprusside
13. Dermatological medicines (topical)	Sodium thiosulfate Terbinafine Mupirocin Potassium permanganate Urea Fluorouracil	
14. Diagnostic agents	Amidotrizoate	Meglumine iotroxate
15. Disinfectants and antiseptics	Glutaral	
16. Diuretics	Amiloride	
18. Hormones, other endocrine medicines and contraceptives	Estradiol cypionate + medroxyprogesterone acetate Diaphragms Levonorgestrel releasing implant Potassium iodide, Propylthiouracil (not available in SA)	
19. Immunologicals	Tuberculin (PPD) Diphtheria antitoxin Cholera vaccine Hepatitis A vaccine Japanese encephalitis vaccine Meningococcal meningitis vaccine Mumps vaccine Rotavirus vaccine Rubella vaccine Typhoid vaccine Yellow fever vaccine	
21. Ophthalmological preparations	Azithromycin Latanoprost	Bevacizumab
23. Peritoneal dialysis solution	Intraperitoneal dialysis solution	
24. Medicines for mental and behavioural disorders	Clomipramine Nicotine replacement therapy	
27. Vitamins and minerals	Ascorbic acid (only available in a multivitamin preparation) Riboflavin (only available in a multivitamin preparation) Sodium fluoride	

Note: The tertiary/quaternary EML was not considered in this analysis

The comparison of medicines for children between the SA STG/EMLs (2008 PHC and 2013 SA Paediatric Hospital STG/EML) and 2012 WHO Model List of PMWC (Table 5) showed some alignment for most conditions as only 3 medicines (of which 2 were antimalarials not available in SA) and 11 of the 20 vaccines were absent.

**Table 5:** Comparison between the WHO PMWC list and SA STG/EMLs

Priority life‐saving medicines for children under five years of age for major causes of mortality and morbidity, palliative care and child survival								
Condition	Drug	Formulation and Strength	PHC 2008		ADULT 2012		PAED 2013	
			Mol	DF	Mol	DF	Mol	DF
Pneumonia	Amoxicillin	dispersible, scored tablets 250 mg and 500 mg or equivalent flexible oral solid dosage form	√	X			√	X
Pneumonia	Ampicillin	powder for injection 500 mg and 1 g	X				√	IV
Pneumonia	Ceftriaxone	powder for injection 250 mg and 1 g	√	IV			√	IV
Pneumonia	Gentamicin	injection 40 mg/ml; 20 mg/ml	X				√	IV
Pneumonia	Oxygen	medicinal gas	√				√	
Diarrhoea	Oral rehydration salts (ORS):	sachets of 200 ml; 500 ml and 1 l, appropriate flavour	√	sachets			√	sachets
Diarrhoea	Zinc	20 mg scored dispersible tablet or equivalent flexible oral solid dosage form	X				X	
Malaria	Artemesinin combination therapy (ACT)	dispersible tablet or flexible oral solid dosage form and dose optimized	X				X	
Malaria	Artesunate	rectal and injection dosage forms 50–200 mg	X				X	
Neonatal sepsis	Ampicillin	powder fir injection 250 mg and 500 mg	X				√	IV
Neonatal sepsis	Ceftriaxone	powder for injection 250 mg and 500 mg	√	IV			√	IV
Neonatal sepsis	Gentamicin	injection 40 mg/ml; 20 mg/ml	X				√	IV
Neonatal sepsis	Procaine benzylpenicillin	powder for injection 1 g	X				√	IM
HIV	Standard regimen for first-line antiretroviral treatment	1 NNRTI + 2 NRTIs e.g. lamivudine + nevirapine + zidovudine - tablet 30 mg + 50 mg + 60 mg; 150 mg + 200 mg + 300 mg	√	oral			√	oral
Vitamin A deficiency	Vitamin A	capsule 100 000 IU strength; 200 000 IU strength	√	capsule			√	capsule
Palliative care/Pain	Morphine	granules 20 mg, 30 mg, 60 mg, 100 mg, 200 mg; injection 10 mg/ml, oral liquid 10 mg/5 ml,	√	IV			√	Oral liquid
Palliative care/Pain	Paracetamol	variable flexible oral solid dosage forms	√	x			√	x
Vaccines	BCG		√				√	
Vaccines	Hepatitis B		√				√	
Vaccines	Polio		√				√	
Vaccines	DTP		√				X	
Vaccines	*Haemophilus influenze* Type b		√				X	
Vaccines	Pneumococcal (conjugate)		X				√	
Vaccines	Rotavirus		X				X	
Vaccines	Measles		√				√	
Vaccines	Rubella		X				X	
Vaccines	HPV		X				X	
Vaccines	Japanese Encephalitis		X				X	
Vaccines	Yellow fever		X				X	
Vaccines	Tick Borne encephalitis		X				X	
Vaccines	Typhoid		X				X	
Vaccines	Cholera		X				X	
Vaccines	Meningococcal		X				X	
Vaccines	Hepatitis A		X				X	
Vaccines	Rabies		√				√	
Vaccines	Mumps		X				X	
Vaccines	*Influenze* (Inactivated)		√				√	
Priority life‐saving medicines for women for major causes of sexual and reproductive health related mortality and morbidity								
Post-partum haemorrhage	Oxytocin	injection 10 IU in 1‐ml ampoule	√		√			
Post-partum haemorrhage	Misoprostol	tablet 200 micrograms (when oxytocin is not available or cannot safely be used)	√		√			
Post-partum haemorrhage	Sodium chloride	injectable solution 0.9% isotonic or	√		√			
Post-partum haemorrhage	Sodium lactate compound	injectable solution (Ringer’s lactate) for infusion	√		X			
Severe pre‐eclampsia/eclampsia	Magnesium sulfate	injection 500 mg/ml in 10‐ml ampoule	√		√			
Severe pre‐eclampsia/eclampsia	Calcium gluconate injection (for treatment of magnesium toxicity)	100 mg/ml in 10‐ml ampoule	√		√			
Severe pre‐eclampsia/eclampsia	Hydralazine	powder for injection 20 mg (hydrochloride) in ampoule or tablet 25 mg; 50 mg (hydrochloride)	X		√			
Severe pre‐eclampsia/eclampsia	Methyldopa	tablet 250 mg	√		√			
Maternal sepsis	Ampicillin	powder for injection 500 mg; 1 g	X		√			
Maternal sepsis	Gentamicin	injection 40 mg/ml in 2‐ml vial	X		√			
Maternal sepsis	Metronidazole	injection 500 mg in 100‐ml vial	X		√			
Abortion/miscarriage	Misoprostol	tablet 200 micrograms	√		√			
Abortion/miscarriage	Mifepristone + misoprostol	tablet 200 mg + tablet 200 micrograms (where permitted under national law)	X		√			
STIs	Azithromycin	capsule 250 mg; 500 mg or oral liquid 200 mg/5 ml	X		X			
STIs	Cefixime	capsule 400 mg	√		√			
STIs	Benzathine Benzylpenicillin	powder for injection 900 mg benzylpenicillin in 5 ml vial; 1,44 g benzylpenicillin in 5 ml vial	√		√			
Preterm labour	Nifedipine	immediate release capsule 10 mg	√		√			
preterm labour (foetal lung maturity)	Dexamethasone	injection 4 mg dexamethasone phosphate (as disodium salt) in 1‐ml ampoule or	X		√			
preterm labour (foetal lung maturity)	Betamethasone	injection 6 mg/ml (3 mg/ml betamethasone sodium phosphate + 3 mg/ml betamethasone acetate) in an aqueous vehicle	√		√			
Maternal Malaria	Artesunate	parenteral	X		X			
Maternal Malaria	Artemether	parenteral	X		X			
Maternal Malaria	Quinine	parenteral	√		√			
Maternal HIV/AIDS	AZT; 3TC; EFV; NVP; EFV; TDF; FTC; ABC; LPV	Doses not stipulated	√		√			
Prevention of tetanus from MTC	Tetanus vaccine		√		√			
Contraception	Oral contraceptives such as ethinylestradiol + levonorgestrel	tablet 30 micrograms + 150 micrograms;	√		√			
Contraception	levonorgestrel	750 micrograms (pack of 2);	√		X			
Contraception	Injectable: estradiol cypionate + medroxyprogesterone acetate	injection 5 mg + 25 mg or	X		X			
Contraception	medroxyprogesterone acetate	depot injection 150 mg/ml in 1‐ml vial or	√		X			
Contraception	norethisterone enantate	200 mg/ml in 1‐ml ampoule	√		X			
Contraception	implantable: levonorgestrel-releasing implant	150 mg total	X		X			
Contraception	intrauterine devices and barrier methods of contraception	copper-containing devices; condoms; diaphragms	√		X			

*Mol* Molecule, *DF* Dosage Form

When compared for medicines for women, the SA STG/EMLs (PHC 2008 and Adult 2012) and WHO list of PMWC seem adequately aligned as only 5 medicines did not appear on either level of SA STG/EML. These included Azithromycin for STIs; Artesunate and Artemether (which are not available in SA) for Maternal Malaria; and 2 medicines for Contraception (injectable oestradiol cypionate + medroxyprogesterone acetate and implantable levonorgestrel-releasing implant.). However, at least one medicine was listed on SA STG/EMLs for each of these categories.

## Discussion

Since inception in 1996, the SA STG/EMLs have undergone substantial transformation in the number of medicines (both molecules and dosage forms) made available with subsequent editions impacting on strengthening the provision for health care in the country. Subsequent to this research a 2014 version of the PHC STG/EML and a 2015 version of the Adult hospital STG/EML have been published by the SA National Department of Health.

### Main findings

There have been many additions and deletions of medicines to the STG/EMLs over time. Possible reasons for deletions include safety and efficacy reasons; adverse effects and/or the availability of safer or cheaper alternatives. For example, the NEMLC secretariat recently issued a notice, to award a tender to a single member in the macrolide class due to cost and clinical advantages (dosing of 24 hourly compared to 6 hourly; improved gastro-intestinal tolerability and consequently improved patient compliance) [22].

Furthermore, the decrease in ratios of molecules between the first and latest STG/EMLs for the section on antineoplastic and immunomodulating agents for both the adult and paediatric hospital level lists may be attributed to the further differentiation of the health care system into levels with the development of a tertiary/quaternary medicine list in 2012, as many of these medicines now appear on this latter list.

Over the years STG/EML changes have increased provision at hospital level for mental health conditions, neonatal conditions, palliative care, HIV/AIDS and related opportunistic infections. Large net changes resulting from the numerous additions and deletions of medicines to each edition of STG/EML were noted for most STG/EMLs that were revised after a period of 5 years (Table 3). During this time lapse there could have been significant changes in the availability of more generics per molecule thus reducing the cost of medicines due to competition in the market. Furthermore, the critical appraisal of evidence could have become more stringent over the years, contributing to numerous additions and deletions in STG/EMLs.

The comparative analysis of the SA STG/EMLs and the WHO PMWC shows that SA STG/EMLs are deficient in the listing of vaccines as 11 out of the 20 vaccines listed on the PMWC list were absent on SA STG/EMLs. In SA the immunization schedule is as per the Expanded Programme on Immunization (EPI-SA), which is outlined in the Vaccinator’s Manual published by the SA National Department of Health [23], which does include the Rotavirus vaccine which is not listed in the SA STG/EMLs (PHC 2008 and Paediatric 2013), though reference to the EPI-SA is made in the STG/EMLs. Vaccines such as Rubella and Mumps as part of the Measles/Mumps/Rubella (MMR) vaccine, HPV, Meningococcal and Hepatitis A are present on the PMWC list but not listed on the EPI-SA and are only available in the private sector of healthcare. These vaccines, for example Rubella, Hepatitis A, and Mumps, with the exception of vaccines for tropical diseases, should be made available in the public sector of healthcare, at the PHC level at the least since this is the first point of contact for most patients in SA. Of the twenty vaccines listed on the PMWC, five are not applicable in SA and are not listed on the EPI-SA (viz. Japanese encephalitis; yellow fever; tick borne encephalitis; cholera and typhoid), and 5 are available in the private sector of healthcare only, which can be considered at a later stage and self-financed. SA did not meet the 2015 MDG target of 20 deaths per 100 000 live births for children under 5 years [7]. Although the comparative analysis in our study shows SA STG/EMLs and the WHO PMWC lists for children are almost aligned for most conditions, this further reiterates the importance for SA to adequately integrate the country’s STG/EMLs with other clusters within SA National Department of Health, especially with regard to vaccines, which could be expanded, at PHC level as these are considered priority life-saving medicines. Further studies are required to analyse the discrepancies between these lists to better describe the impact of the differences on improving the under-5 mortality.

The previous increase in the under-5 child morbidity could also be linked to other barriers to access to healthcare. A study conducted by Lalloo et al. (2004) reported that although policies aimed at improving healthcare and reducing inequities in access to health care were implemented, these inequities still existed and over one quarter of SA citizens were unable to access health care [24].

Differences between SA STG/EMLs and WHO Model EMLs are expected since the WHO Model EML is merely a guideline for the development of EMLs globally. Possible reasons for discrepancies may include a medicine or vaccine not being considered essential to the burden of disease for SA; or differences in recommended therapy between national and WHO guidelines; or certain medicines listed on the WHO EML may not be licenced for use in SA [25]. The arrangement of the WHO Model EMLs according to pharmacological class is different to that of the SA STG/EMLs which is arranged according to organ systems. These may also be possible reasons for differences between SA and studies in other countries.

### Comparison with other studies

Similar studies to assess changes in countries’ EMLs and compare alignment of lists with WHO EML have been conducted in other countries [26–29]. This is the first analysis to report changes in SA STG/EMLs in terms of molecules, dosage forms and therapeutic classes. The paucity of reports on STG/EML changes, together with supporting documents detailing reasons for changes, by the NEMLC hinders an in-depth analysis. This is in direct contrast to the more transparent reporting process followed by the WHO, whereby all information pertaining to the rationale and cited evidence for each change (addition or deletion) is captured and subsequently published as a Technical Report Series document. In previous years the SA STG/EMLs have been published without these details, however more recently, when chapters from a draft STG/EML are distributed for comment, an accompanying document details the reasoning and lists the evidence cited.

A comparative analysis of the WHO Model EML by year since 1977 was conducted [10, 11] to describe the process of development and updating of the list over time. The importance of supporting evidence for the assessment of safety, comparative effectiveness, and cost of medicines was emphasized. The study showed an increase in the overall number of molecules including duplicates (216 to 423) and the ratio between formulations/dosage forms and molecules from 0 to 1.9 from the first edition in 1977 to 2009, indicating a similar trend to this study.

A study conducted in Croatia used the WHO Model EML to assess the appropriateness of insurance coverage decisions for the 2010 insurance coverage list compiled by the Croatian Institute for Health Insurance (CIHI). The study compared medicines on the CIHI Basic List with the WHO EML and found 188 medicines in common. The WHO EML contained 32 individual and 10 combinations of the medicines which were absent on the CIHI Basic List. Most differences were noted for infectious diseases medicines [26]. This is fewer than the SA STG/EMLs which had 101 individual and 11 combinations of medicines that were absent but was similar in that it also vastly differed in the anti-infectives medicines category.

China has implemented an EML since 1982 and in March 2013 released the new revised 2012 EML. China’s EML contains both Western and Traditional Chinese medicines. Zang et al., (2003) analysed the new EML and found an increase in total number of western molecules (307 to 520) and number of formulations (780+ to 850+) from 2009 to 2012. China’s Ministry of Health also hope to implement policies on restriction of medicine utilization to EML items only for all government-based PHC institutes; alignment of procurement policies for EML items only; and make changes in the EML tendering mechanisms [27]. China’s increasing trend in number of molecules and formulations is similar to the increasing trend noted in SA STG/EMLs. Furthermore, a comparison of China’s 2009 EML with WHO EML showed that 132 medicines on the WHO EML were absent from China’s EML [28] which is almost similar to the number of absent medicines on the SA STG/EMLs in this study.

Tejani and Wertheimer (2014) analysed EMLs of ten developing nations in Africa (excluding SA) and found that most countries had on average 237 medications on their EMLs compared to 350 on the 2013 WHO Model EML. Eritrea had the most number of medications (354) and Somalia the least (85). When compared to the WHO EML, on average, most countries had 58.23% of medicines in common, were lacking approximately 47% of medicines, and had 30% additional medicines on their EMLs. This comparison indicated that at least 18 medicines were common among the countries (and not present on the WHO EML) [29]. In comparison, our study showed that SA had 112 absent medicines (excluding duplicates).

In 2012, Hill et al. performed a global survey of 89 country EMLs for inclusion of WHO PMWC (SA not included). The most commonly listed medicines were as follows: paracetamol (appeared on 94% of EMLs); Sodium chloride, oral rehydration solution and gentamicin (93%). Least commonly listed were children’s antimalarials (rectal artesunate (8%); artesunate injection (16%); Paediatric artemisinin combination therapy (36%); Procaine benzylpenicillin (50%) and Zinc (15%). Analysis of medicines for women showed the following: oxytocin (appeared on 62% of EMLs); Magnesium sulphate (50%); misoprostol (35%); and cefixime (26%). Our study showed similar results in listing of common medicines for both mothers and children and also did not list similar children’s medicines since certain medicines (such as artesunate and artemisinin combination therapy) as well as certain dosage forms (such as dispersible, scored tablets for zinc, amoxicillin and paracetamol) are not available in SA.

### Limitations

This study did not consider SA’s Gross Domestic Product, the budget for medicines, and the specific disease burden for the country. It also did not examine the criteria and processes for medicines selection for national EMLs. This study also did not assess the impact on health outcomes by implementation and changes in SA STG/EMLs over time. These pose as limitations in this study.

In addition, during the data analysis it was noted that the index of drugs in the various SA STG/EMLs are not complete, comprehensive listings of essential medicines. Medicines that were mentioned in the STG were sometimes absent in the index of drugs. In addition, some medicines were listed as their common names and some were incorrectly spelt. This hinders the ability to quickly find medicines and related treatment. Furthermore, a complete listing of registered drugs in SA is not readily available.

### Suggestions for future research

More research is required into the decision-making process that the committees engage in during the review process and on the impact of therapeutic class selection in the procurement and use of medicines processes as well as whether regular review processes impact on STG/EML use and acceptance.

There is a dearth of published information on the NEML changes (until just recently by the secretariat within the National Department of Health), with regard to the reasons for addition and deletion of medicines with supporting evidence; processes and procedures of the NEMLC regarding such changes and underlying policies employed; and reports on implementation, monitoring and evaluation of STG/EMLs in SA. Thus, these are recommendations for future research and for the workings of the NEMLC to move towards a more transparent, frequent reporting system as an approach to strengthen the provision of health care in the country and also impacting on improving access to pharmaceuticals and services from a pharmaceutical policy point of view since NEMLC decisions feed into the tendering and subsequent procurement processes of pharmaceuticals.

SA has moved towards different levels of care. The EML changes described in this study indicates the drive SA has to define these various levels of care by introducing prescriber levels, task shifting and the decentralization of ARV services to PHC [19], and the production of a tertiary/quaternary list, all of which aim to improve the provision of healthcare in the public sector in SA.

### What this study adds and implications for the SA process

Monitoring and evaluation of essential medicines policies, its implementation and outcomes is essential to improve the efficiency and effectiveness of an EMP. Regular assessment of the policy’s performance will provide insight into the costs, results and value of the programme which ultimately informs the policy decision making process and overall management of the policy and programme. The SA STG/EMLs have been implemented for a few years now and there has been no analysis of this nature previously The analysis in this study is the first of this kind and although it has revealed changes, it is difficult to assess if these changes were rational nor are the underlying reasons for changes transparent. Thus this study calls for continuous monitoring of STG/EML changes.

## Conclusions

This is the first study in SA to investigate changes in NEMLs over time in relation to molecules, dosage forms and therapeutic classes. It is also the first to compare the latest SA EMLs to the WHO Model lists. The results therefore provide insight into the SA EML processes over time.

It is important to continue to monitor STG/EML development and implementation, at both national and provincial PTC levels, in order to understand the reasons behind changes so as to adequately address the healthcare needs of the population within budgetary constraints of a country. The concept of essential medicines has gained momentum worldwide, and evidence is required to ascertain whether such a policy does make a difference in outcomes for healthcare systems and patients. It is important to note that this study did not look at decision making processes for EML selection in the STG development process. From the changes that have been described, there might have been some principles employed in decisions. However, further studies are required to confirm this.

## Abbreviations

CIHI: Croatian institute for health insurance
EML: Essential medicines list
EMP: Essential medicines programme
EPI: Expanded programme on immunization
MDG: Millennium development goals
NDP: National drug policy for South Africa
NEML: National essential medicines list
NEMLC: National essential medicines list committee
PHC: Primary health care
PMWC: Priority life-saving medicines for women and children
PTC: Pharmacy and therapeutics committee
SA: South Africa
SDG: Sustainable development goals
STG: Standard treatment guidelines
WHO: World Health Organization

## Footnotes

1 This reference refers to the medicine procurement list which is constantly updated and the most recent master procurement list was reflected.
